# IL-21 Receptor Antagonist Inhibits Differentiation of B Cells toward Plasmablasts upon Alloantigen Stimulation

**DOI:** 10.3389/fimmu.2017.00306

**Published:** 2017-03-20

**Authors:** Kitty de Leur, Frank J. M. F. Dor, Marjolein Dieterich, Luc J. W. van der Laan, Rudi W. Hendriks, Carla C. Baan

**Affiliations:** ^1^Department of Internal Medicine, Erasmus MC, University Medical Center, Rotterdam, Netherlands; ^2^Division of HPB and Transplant Surgery, Department of Surgery, Erasmus MC, University Medical Center, Rotterdam, Netherlands; ^3^Department of Pulmonary Medicine, Erasmus MC, University Medical Center, Rotterdam, Netherlands

**Keywords:** alloreactivity, B cell differentiation, plasmablast, IL-21 receptor, follicular T-helper cell

## Abstract

Interaction between T follicular helper (Tfh) cells and B cells is complex and involves various pathways, including the production of IL-21 by the Tfh cells. Secretion of IL-21 results in B cell differentiation toward immunoglobulin-producing plasmablasts. In patients after kidney transplantation, the formation of alloantibodies produced by donor antigen-activated B cells are a major cause of organ failure. In this allogeneic response, the role of IL-21-producing Tfh cells that regulate B cell differentiation is unknown. Here, we tested, in an alloantigen-driven setting, whether Tfh cell help signals control B cell differentiation with its dependency on IL-21. Pre-transplantation patient PBMCs were sorted into pure CD4^pos^CXCR5^pos^ Tfh cells and CD19^pos^CD27^pos^ memory B cells and stimulated with donor antigen in the presence or absence of an IL-21 receptor (IL-21R) antagonist (αIL-21R). Donor antigen stimulation initiated expression of the activation markers inducible co-stimulator (ICOS) and programmed death 1 (PD-1) on Tfh cells and a shift toward a mixed Tfh2 and Tfh17 phenotype. The memory B cells underwent class switch recombination and differentiated toward IgM- and IgG-producing plasmablasts. In the presence of αIL-21R, a dose-dependent inhibition of STAT3 phosphorylation was measured in both T and B cells. Blockade of the IL-21R did not have an effect on PD-1 and ICOS expression on Tfh cells but significantly inhibited B cell differentiation. The proportion of plasmablasts decreased by 78% in the presence of αIL-21R. Moreover, secreted IgM and IgG2 levels were significantly lower in the presence of αIL-21R. In conclusion, our results demonstrate that IL-21 produced by alloantigen-activated Tfh cells controls B cell differentiation toward antibody producing plasmablasts. The IL-21R might, therefore, be a useful target in organ transplantation to prevent antigen-driven immune responses leading to graft failure.

## Introduction

After kidney transplantation, the immunological barrier between organ donor and recipient still limits graft survival (1). In this setting, a large proportion of allograft recipients develop a donor-specific antibody response associated with an increased risk for chronic rejection (2–5). This complication accounts for more than 50% of chronic transplant failures leading to death, dialysis, or re-transplantation of patients (3). Current immunosuppressive agents mainly aimed at T-cell-mediated alloimmunity, whereas agents that effectively target humoral effectors are still insufficient (6). Therefore, there is a need to develop new agents that specifically prevent the activation of B cell-mediated immune responses.

Within humoral immunity, T cell-mediated help to B cells is required for the generation of antigen-specific antibody responses. This process is mainly driven *via* IL-21-secreting T follicular helper (Tfh) cells. Tfh cells are well known for their expression of CXC chemokine receptor 5 (CXCR5) (7). Sustained expression of CXCR5 helps Tfh cells localize to B cell follicles, where they interact with germinal center (GC) B cells and produce IL-21 (8). Through autocrine and paracrine mechanisms, IL-21 amplifies and stabilizes Tfh cell-mediated responses, B cell proliferation, immunoglobulin class switch recombination (CSR), and B cell differentiation toward plasmablasts and long-living memory B cells (9, 10). In this respect, IL-21 directly effects B cell responses *via* IL-21 receptor (IL-21R) expressed on the B cells (11, 12). IL-21 signals through a receptor complex consisting of IL-21R and a common cytokine receptor γ-chain that activates downstream JAK/STAT pathways, predominantly by the phosphorylation of STAT3 (13, 14). Transcriptional repressor B-cell lymphoma 6 (Bcl-6) orchestrates the differentiation program of Tfh cells, while suppressing other T helper subset transcription factors (8, 15). The capacity of Tfh cells to interact with B cells is dependent on T-cell receptor interaction with antigens presented by MHC class II molecules and co-stimulatory molecules CD40ligand, inducible co-stimulator (ICOS), and programmed death 1 (PD-1) (7, 8). The circulating counterparts of the “GC-Tfh cells” in humans express CXCR5, low expression levels of PD-1 and ICOS and lack expression of transcription repressor Bcl-6 (16–18).

In transplantation, studies on peripheral Tfh cells and their role in IL-21 driven B cell differentiation are limited (19, 20). An increased frequency of circulating Tfh cells was found in patients with chronic antibody-mediated allograft rejection after kidney transplantation (21). Furthermore, in patients with pre-existing donor-specific antibodies (DSA), an association was detected between pre-existing DSAs and the numbers of Tfh cells after transplantation (22). Co-stimulation blockade in a non-human primate kidney transplant model resulted in reduced IL-21 production in GC and an attenuated antibody response (23). In addition, selective blockade of CD28 solely resulted in lower levels of IL-21 compared to CD80/86 co-stimulatory blocking therapy (24) For the development of immunosuppressive agents that specifically target B cell-mediated immune responses directed toward donor antigen early in the activation cascade, a better understanding of Tfh biology is needed.

Kidney disease patients suffer from defective immune responses caused by decreased T and B cell activity (25, 26). Therefore, we have used patient materials to set up an *in vitro* system in which we studied whether Tfh cells instruct donor antigen-driven memory B cells to differentiate into immunoglobulin producing plasmablasts. Subsequently, we assessed whether this Tfh cell-mediated differentiation and plasmablast formation is dependent on IL-21 by blocking the IL-21R with an antagonist (αIL-21R). Overall, our data define the role of IL-21/IL-21R signaling pathway in alloantigen-driven and Tfh cell-mediated B cell differentiation toward Ig-producing plasmablasts.

## Materials and Methods

### Study Population

For the *in vitro* assays, PBMCs of 17 kidney transplant recipients obtained 1 day pre-transplantation were analyzed and stimulated with the corresponding kidney donor PBMCs. Patient demographics are summarized in Table 1. The Medical Ethical Committee of the Erasmus MC, University Medical Center, approved this study (MEC-2010-022). All patients and donors gave written informed consent. B cell parameters were measured in all samples and T cell assays were performed when enough material was available for analysis.

**Table 1:** **Patient characteristics at baseline**.

	Study group (*n* = 17)
Patients age in years (median, range)	57 (33–74)
Recipient gender (% male)	76.5%
HLA-A mismatches (mean ± SD)	1.1 (±0.7)
HLA-B mismatches (mean ± SD)	1.7 (±0.5)
HLA-DR mismatches (mean ± SD)	1.5 (±0.5)
Panel reactive antigen (median, range)	
• Current	0.0% (0.0–71%)
• Peak	4.0% (0.0–99%)
Previous kidney transplantation	11.8% (2)
• Second kidney transplantation	5.9% (1)
• Third kidney transplantation	5.9% (1)
Renal replacement therapy before transplantation	88.3% (15)
• Hemodialysis	76.5% (13)
• Peritoneal dialysis	11.8% (2)
Cause of end-stage renal disease	
• Hypertensive nephropathy	35.3% (6)
• Diabetic nephropathy	41.2% (7)
• Focal segmental glomerulosclerosis	5.9% (1)
• IgA nephropathy	5.9% (1)
• Polyarteritis nodosa	5.9% (1)
• Unknown	5.9% (1)

*Numbers inside the brackets represent patient number unless otherwise specified*.

### Coculture Experiments of Peripheral Tfh Cells and Memory B Cells

Coculture experiments with Tfh cells and memory B cells were conducted as schematically represented in Figure S1 in Supplementary Material to determine the functional interactions after donor antigen stimulation. PBMCs were thawed and CD3^pos^CD4^pos^CXCR5^pos^ T cells (Tfh cells) and CD19^pos^CD27^pos^ (memory) B cells were fluorescence activated cell sorted by BD-FACSAria II SORP™ (purities ≥96%). Isolated Tfh cells (2 × 10^4^ cells/well) were cocultured with memory B cells (2 × 10^4^ cells/well) in a 96-well plate for 8 days in the presence of irradiated (40 Gy) donor PBMCs (5 × 10^4^/well). At day 0 and after 8 days of culture, the Tfh cell phenotype, the B cell phenotype, and B cell differentiation toward Ig-producing plasmablasts were measured with flow cytometry. The following monoclonal antibodies (MoAbs) were used for the Tfh cell phenotype stainings: CD3 Brilliant Violet 510 (BV510) (Biolegend, San Diego, CA, USA), CD4 Brilliant Violet 421 (BV421, Biolegend), CXCR5 Alexa Fluor 647 (AF647, BD Biosciences, San José, CA, USA), ICOS phycoerythrin-Cyanine7 (PE-Cy7, Biolegend), CCR6 PE (eBioscience, San Diego, CA, USA), CXCR3 peridinin chlorophyll (PerCP, Biolegend), and PD-1 Allophycocyanin-Cy7 (APC-Cy7, Biolegend). MoAbs used for the B cell stainings: CD19 BV510 (Biolegend), CD38 BV421 (BD Biosciences), IgG APC (BD Biosciences), CD27 Pe-Cy7 (eBioscience), IgM PE (BD Biosciences), and IgD APC-Cy7 (Biolegend). 7-aminoactinomycin (7-AAD) PerCP was included to measure cell viability. To define the role of IL-21/IL-21R signaling in alloantigen-activated Tfh and memory B cells, the cocultures were pre incubated for 30 minutes with humanized anti-IL-21R antibody ATR-107 (10 μg/ml, kindly provided by Pfizer, New York, NY, USA) or isotype-matched control (10 μg/ml, IgG1-Fc, R&D systems, Minneapolis, MN, USA) at 37°C. Hereafter, the irradiated donor cells were added to the cocultures and further incubated at 37°C for 8 days. Total IgM, IgG, and IgG2 production was measured in the culture supernatants with a sandwich ELISA (eBioscience). All flow cytometry analyzes were performed with Kaluza Analysis 1.3 (Beckman Coulter, Fullerton, CA, USA).

### Phospho-Specific Flow Cytometry

Phosphorylation of STAT3 by CD4^+^ T cells and CD19^+^ B cells was determined by phospho-specific flow cytometry. In brief, PBMCs were stained with CD3 BV510 (Biolegend) and CD19 Pe-Cy7 (Biolegend) for 30 min at RT in the dark. Next, the cells were incubated for 30 min at 37°C with various concentrations of the humanized anti-IL-21R antibody ATR-107 (Pfizer) or isotype-matched control (IgG1-Fc, R&D systems) followed by stimulation with recombinant human IL-21 (100 ng/ml, eBioscience) or recombinant human IL-6 (100 ng/ml, PeproTech, Rocky Hill, NJ, USA) for 15 min at 37°C. Cells were fixed for 10 min with Cytofix buffer (BD Biosciences) at 37°C and permeabilized 30 min in 1 ml methanol 90% at −20°C. Next, samples were stained with CD4 BV421 (Biolegend) and pSTAT3 PE (BD Biosciences). STAT3 phosphorylation was calculated as the median fluorescence intensity.

### B Cell Stimulation Assay

B cell stimulation with a minor cocktail of stimuli was performed to study the effect of IL-21, co-stimulation, and BCR activation on plasmablast formation. CD19^+^ B cells were isolated *via* CD43 negative selection with CD43 MicroBeads (Miltenyi Biotec, Bergisch Gladbach, Germany) (purities ≥85%). B cells were incubated with anti-IL-21R antibody ATR-107 (10 μg/ml, Pfizer) or isotype-matched control (10 μg/ml IgG1-Fc, R&D systems). Next, cells were stimulated with 5 μg/ml soluble anti-CD40 (Bioceros, Utrecht, The Netherlands), 10 μg/ml goat-anti-human IgM (Jackson Immunoresearch, West Grove, PA, USA) and human recombinant IL-21 (100 ng/ml, eBioscience). Subsequently, the presence of plasmablasts on day 0 and the differentiation of memory B cells into plasmablasts on day 8 were determined with flow cytometry. Plasmablasts were defined as CD19^pos^CD27^high^CD38^high^ cells (16). The following MoAbs were used: CD19 BV510 (Biolegend), CD27 Pe-Cy7 (eBioscience), IgD APC-Cy7 (Biolegend), and CD38 BV421 (BD Biosciences). In addition, viability staining with 7-AAD PerCP was performed (BD Biosciences).

### Statistics

Statistical analyses were performed using Wilcoxon signed-rank test and Spearman’s rank correlation test using GraphPad Prism 5 software (GraphPhad Software, San Diego, CA, USA, http://www.graphpad.com). A two-tailed *p*-value < 0.05 was considered statistically significant.

## Results

### Tfh Cells Are Activated upon Stimulation with Alloantigen

**Figure 1:**
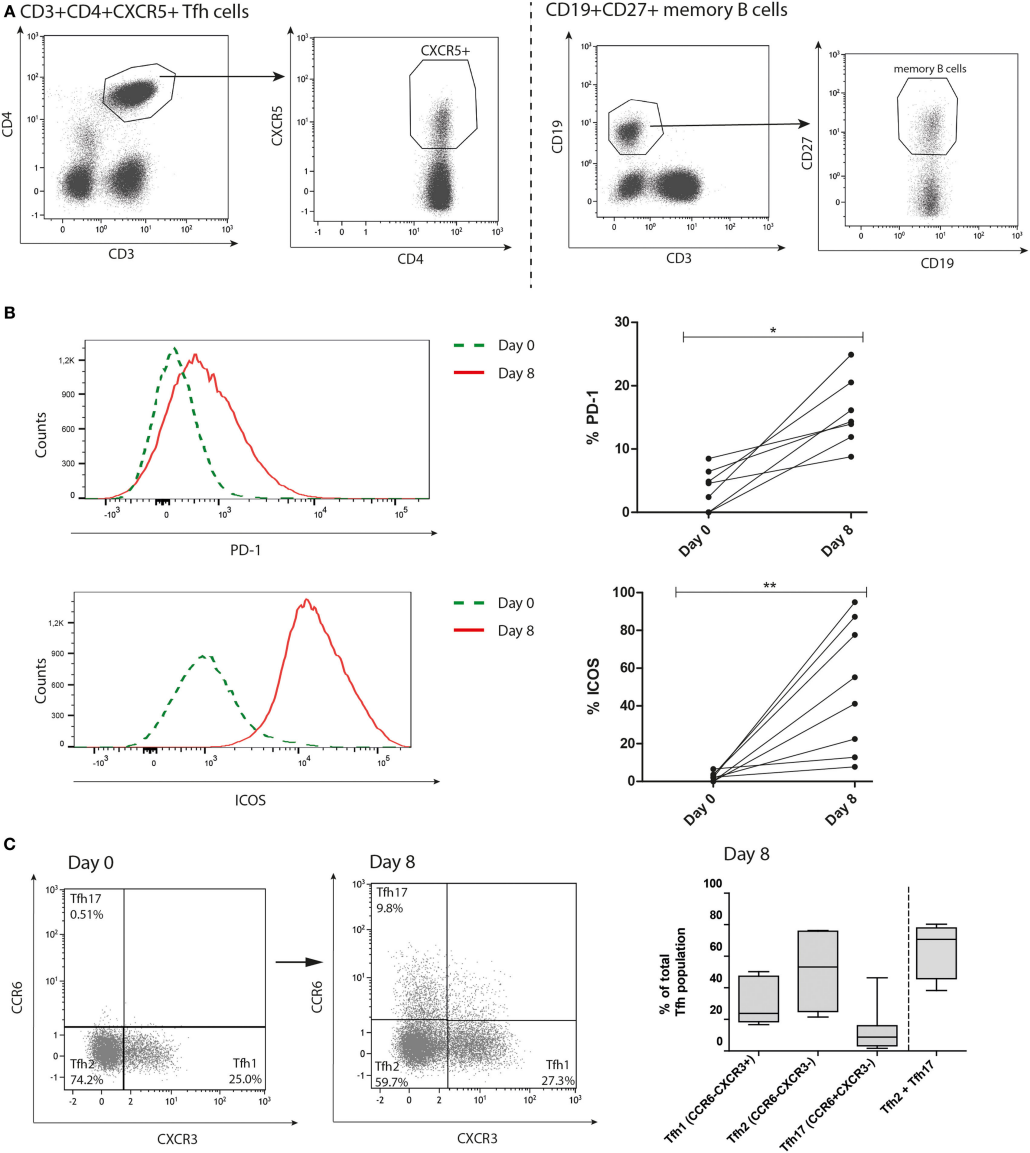
**T follicular helper (Tfh) cells are activated upon stimulation with alloantigen**. Tfh cells and memory B cells from patients, pre kidney transplantation, were stimulated with alloantigen and cocultured for 8 days. **(A)** Typical example of Fluorescence activated cell sorting gating strategy to obtain CD4^pos^CXCR5^pos^ Tfh cells and CD19^pos^CD27^pos^ memory B-cells. Cells were gated from viable (7-AAD negative) lymphocytes, defined by forward- and side-scatter. **(B)** Histogram overlays and quantified data of proportions programmed death 1 (PD-1) and inducible co-stimulator (ICOS) within CD4^pos^CXCR5^pos^ Tfh cells at baseline (day 0) and day 8 after coculture (PD-1 *n* = 7; ICOS *n* = 8). **(C)** Gating strategy and distribution of proportions CCR6^neg^CXCR3^pos^ Tfh1, CCR6^neg^CXCR3^neg^ Tfh2 and CCR6^pos^CXCR3^neg^ Tfh17 cells of the total Tfh population on day 0 and 8 after coculture. N.B.: box whiskers represent minimal and maximal values. The upper and lower borders of the box represent the 25 and 75% percentile, respectively, with the middle line representing the median (*n* = 8).

We set up an *in vitro* system to study the functional interaction of CD4^pos^CXCR5^pos^ Tfh cells and CD19^pos^CD27^pos^ memory B cells upon alloantigen stimulation and the role of IL-21 in this response. Purified Tfh cells and memory B cells were cocultured and stimulated with donor alloantigen. As a negative control, donor alloantigen was omitted (Figure S2 in Supplementary Material). For gating strategies see Figure 1A. Proportions of activation markers PD-1 and ICOS on the Tfh cells significantly increased after coculture (Figure 1B, *p* = 0.02 and *p* = 0.008, respectively).

A heterogeneous Tfh cell population was observed at day 8 based on expression of chemokine receptors CCR6 and CXCR3. Three Tfh subsets can be distinguished: CXCR3^pos^CCR6^neg^ Tfh1 cells, CXCR3^neg^CCR6^neg^ Tfh2 cells and CXCR3^neg^CCR6^pos^ Tfh17 cells. Especially the Tfh2 and Tfh17 subsets (CXCR3^neg^) are able to induce B cell differentiation and Ig CSR *via* IL-21, while the CXCR3^pos^ Tfh1 cells lack this capacity (16, 27). The mean proportion of Tfh2 and Tfh17 cells at day 8 after coculture was 71% (range: 38–80%), forming the majority of the different subsets (Figure 1C).

### Tfh-Mediated B Cell Differentiation Occurs upon Stimulation with Alloantigen

**Figure 2:**
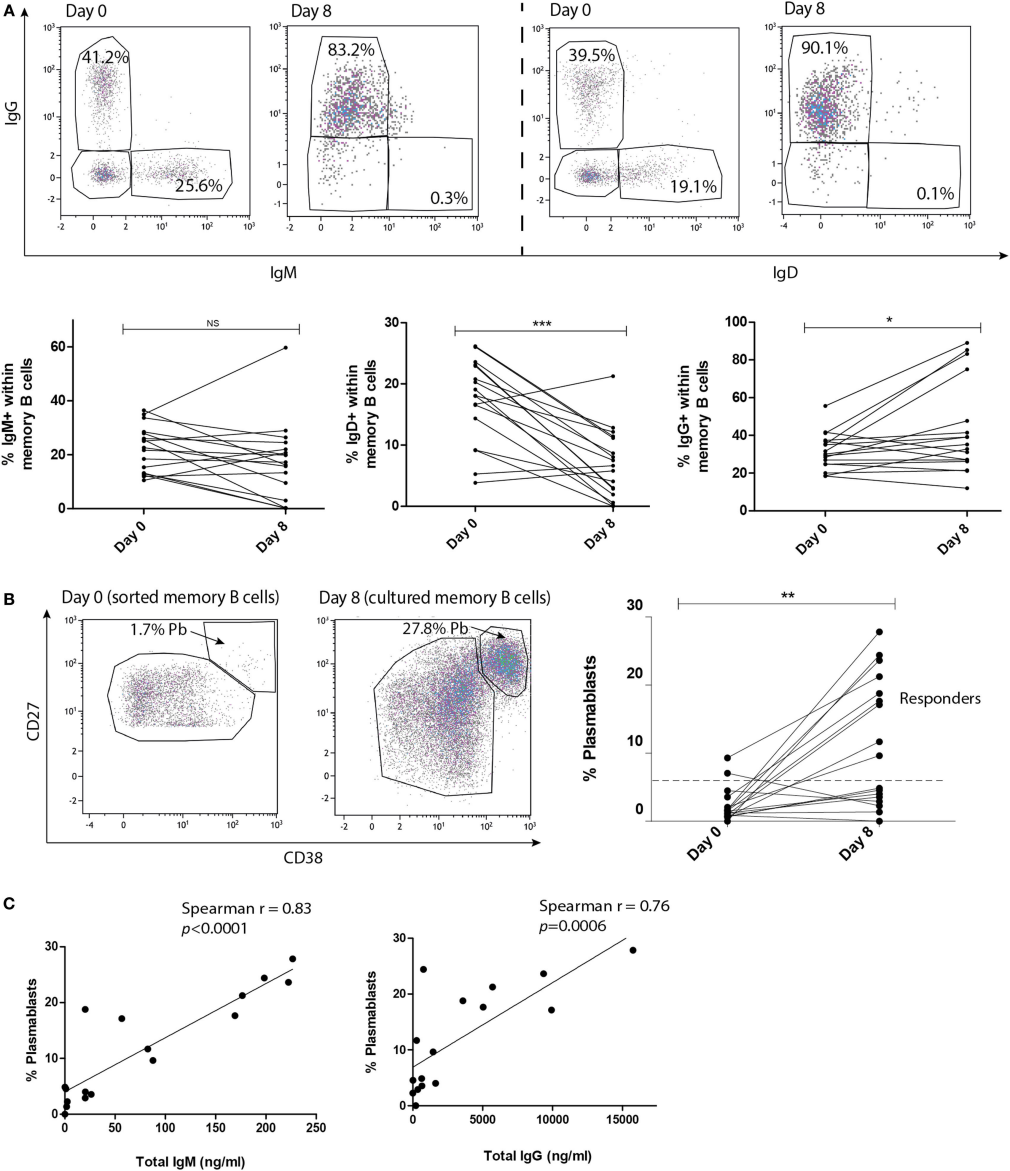
**T follicular helper (Tfh)-mediated B cell differentiation occurs upon stimulation with alloantigen**. Tfh and memory B cells were stimulated with alloantigen and cocultured for 8 days. **(A)** Gating strategy of surface immunoglobulins IgM, IgD, and IgG within the memory B cell population before and after coculture and corresponding quantified data (*n* = 17). **(B)** Gating strategy of CD27^high^CD38^high^ plasmablasts and remaining memory B cells at baseline (day 0) and after 8 days coculture is depicted on the left. Quantified data of plasmablast proportions at day 0 and after 8 days coculture are depicted on the right. Dashed line distinguishes between cultures with a high plasmablast proportion (>10%) compared to a low plasmablast proportion (<5%) at day 8 (*n* = 17). **(C)** Correlation between proportion of plasmablasts and IgM or IgG (ng/ml) in the culture supernatant after 8 days coculture (*n* = 16) (*p < 0.05, **p < 0.003, ***p < 0.0009). Pb, plasmablast.

Over the 8 days of coculture, the composition of surface immunoglobulins on the memory B cells changed. After coculture CSR toward IgG occurred in part of the samples, with mean proportion surface IgG of 30% (range: 18–56%) before coculture toward 35% (range: 12–89%) after coculture (Figure 2A, *p* = 0.04) while IgD proportions decreased from mean proportion of 18% (range: 4–26%) to 7% (range: 0.0–21%) after coculture (Figure 2A, *p* = 0.0009). The proportion of B cells expressing IgM did not significantly change after the coculture (Figure 2A, *p* = 0.09). A significant increase in plasmablast numbers was observed after coculture, proving the robust capacity of alloantigen and Tfh cells in stimulating memory B cell differentiation (Maximum 28%, Figure 2B, *p* = 0.003). This increase in plasmablast numbers was not observed in cocultures without donor alloantigen stimulation (Figure S2 in Supplementary Material). Based on the median proportion (10%) at day 8, we observed a group of cultures with high plasmablast proportions (> 10%, *n* = 9) and a group with low plasmablast proportions after culture (< 5%, *n* = 8). No significant differences were found between the two groups based on baseline characteristics (Table S1 in Supplementary Material). The immunoglobulin producing capacity of the generated plasmablasts was proven by the correlation between the proportion of plasmablasts and the concentration of IgM or IgG in the supernatants of all cultures (Figure 2C, *p* < 0.0001 and *p* = 0.0006, respectively). Overall, we showed that kidney transplant patient-derived circulating Tfh cells before transplantation can be activated and are capable to stimulate memory B cell CSR and differentiation toward plasmablasts in the presence of alloantigen.

### Phosphorylation of STAT3 Is Inhibited in the Presence of αIL-21R Antibodies

**Figure 3:**
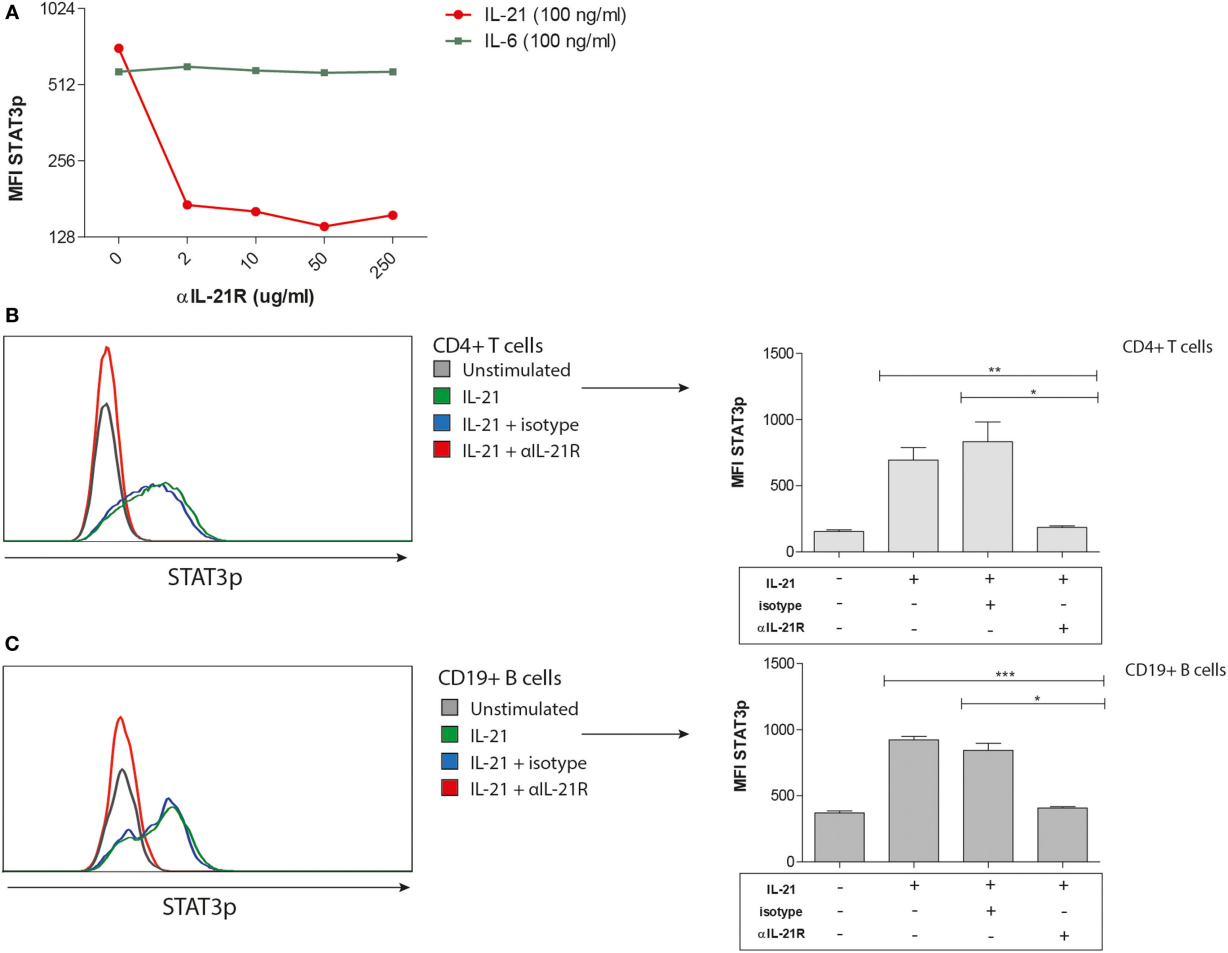
**Phosphorylation of STAT3 is inhibited in the presence of αIL-21R antibodies**. **(A–C)** PBMCs were stimulated with 100 ng/ml IL-21 or 100 ng/ml IL-6 for 15 min in the absence or presence of 10 μg/ml αIL-21R or 10 μg/ml IgG1 isotype. median fluorescence intensity (MFI) values of phosphorylated STAT3 were measured directly afterward. **(A)** Dose–response curve of STAT3p in CD4^pos^ T cells stimulated with 100 ng/ml IL-21 and different concentrations of αIL-21R. Stimulation with 100 ng/ml IL-6 was used as a positive control. **(B–C)** Histogram overlays and quantified data of phosphorylated STAT3 MFI values in CD4^pos^ T-cells **(B)** and CD19^pos^ B-cells **(C)** (*n* = 7) (*p < 0.05, **p < 0.005, ***p < 0.0005). Upper line of the boxes represent mean with SEM represented by the whiskers.

To determine the functionality of the IL-21R antibody (αIL-21R) in blocking IL-21R induced signaling events in T and B cells, we performed phospho-specific flow cytometry. Phosphorylation of STAT3 was measured after stimulation with IL-21 or IL-6 (positive control). The specificity of αIL-21R was demonstrated by a 3.5-fold reduction in the phosphorylation of STAT3 on CD4^+^ T cells in the presence of 2 μg/ml αIL-21R onward when comparing the IL-21 stimulated cells with the IL-6 stimulated cells (Figure 3A). Complete inhibition of STAT3 phosphorylation in the presence of αIL-21R was seen on both T and B cells (Figures 3B,C, *p* = 0.002 and *p* = 0.0005, respectively). In the presence of an isotype-matched control of αIL-21R the STAT3p levels were similar to the condition with only IL-21 stimulation. From these findings we conclude that αIL-21R efficiently blocks IL-21R induced signaling in T and B cells.

### αIL-21R Inhibits Memory B Cell Differentiation upon Alloantigen Stimulation

To explore the importance of the IL-21/IL-21R signaling pathway in an allogeneic system, αIL-21R was added to the cocultures containing Tfh cells and memory B cells stimulated with alloantigen. Both cell populations expressed IL-21R (Figure S3 in Supplementary Material). Prior to the cocultures, we tested the capacity of αIL-21R to block the formation of plasmablasts in a culture system described by Ettinger and colleagues, where B cells were stimulated with anti-CD40, anti-IgM and IL-21 resulting in robust plasmablast proportions (28). Significant inhibition of plasmablast formation was found in the presence of αIL-21R in this setting (Figure S4 in Supplementary Material).

Next, we tested the efficacy of αIL-21R to block in our alloantigen Tfh–B cell coculture system. To determine the inhibitory capacity of αIL-21R on B cell function, we focused on the cultures with the highest proportion of plasmablasts. Cultures with low plasmablast proportions (<5%) were excluded. In these samples, after the culture period of 8 days, no differences were found regarding the phenotype of the Tfh cells. Moreover, proportions of PD-1, ICOS, and the distribution of CCR6 and CXCR3 expressed were comparable in the presence and absence of αIL-21R (Figure S5 in Supplementary Material). Thus, activated Tfh cells and their Tfh1, Tfh2, or Tfh17 phenotype are not altered in terms of their phenotype when cultured with αIL-21R.

**Figure 4:**
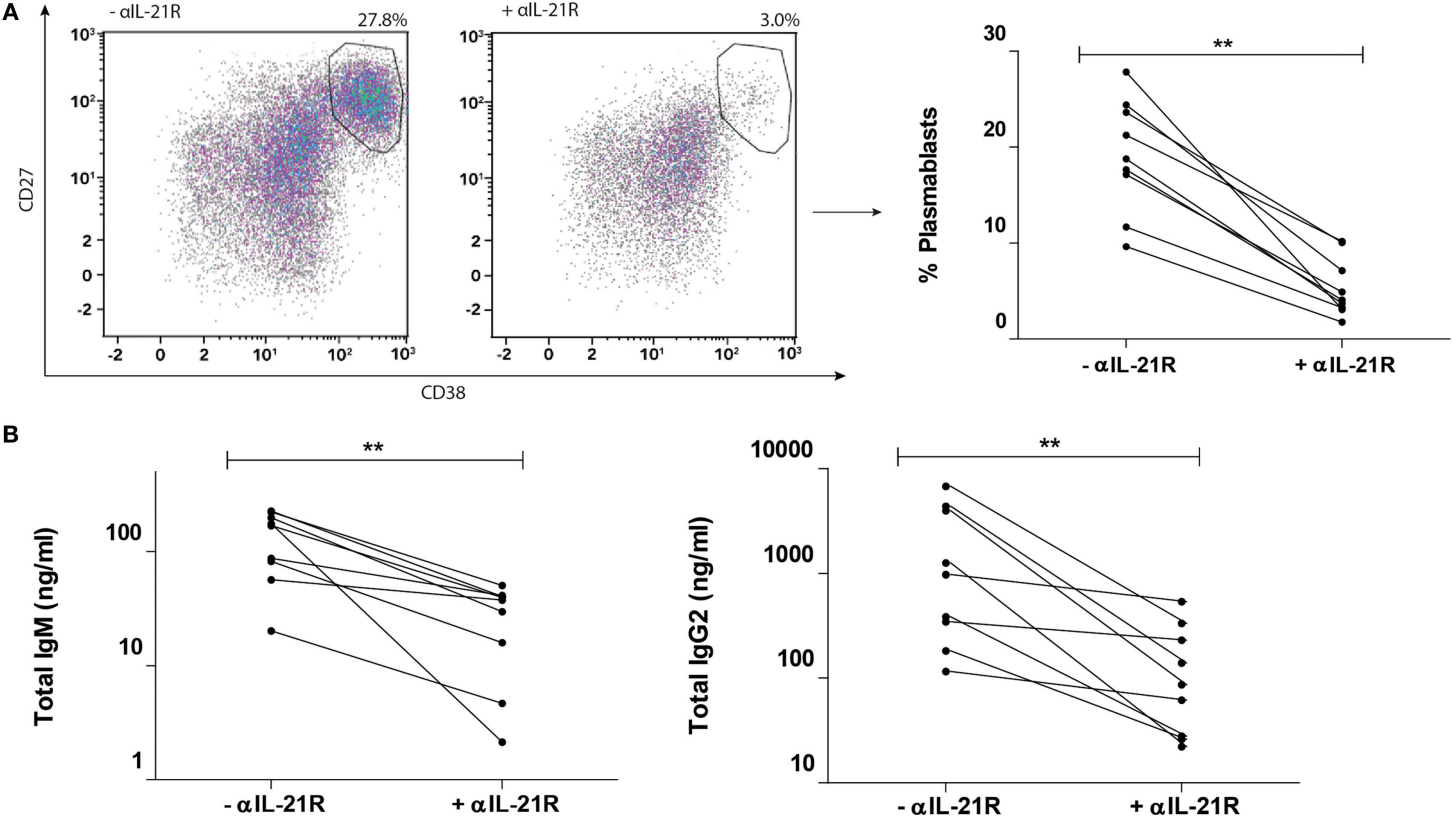
**αIL-21R inhibits memory B cell differentiation upon alloantigen stimulation**. CD4^pos^CXCR5^pos^ Tfh-cells and CD19^pos^CD27^pos^ memory B-cells stimulated with alloantigen were cocultured for 8 days in the presence or absence of 10 μg/ml αIL-21R. Only the cultures with >10% plasmablasts at day 8 (high responders) are depicted. **(A)** Representative dotplots and quantified data of proportions CD27^high^CD38^high^ plasmablasts at day 8 after coculture in the presence or absence of αIL-21R. **(B)** Total IgM and IgG2 measured in supernatants after 8 days coculture (*n* = 9) (**p < 0.004). *y*-axes for Ig production are scaled log-linearly.

In the presence of αIL-21R, formation of plasmablasts was inhibited by 78% (*p* = 0.004, Figure 4A). In parallel, IgM production was inhibited from mean production of 169 ng/ml (range: 20–226 ng/ml) to 38 ng/ml (range: 2–50 ng/ml) in the presence of αIL-21R (*p* = 0.004, Figure 4B). Since αIL-21R is a fully humanized IgG1 compound, we measured IgG2 levels in the culture supernatant. Although IgG2 is a non-complement-fixing antibody, the IgG2 subclass of antibodies is the second most common Ig subclass present after immunization (29). Production of IgG2 decreased from mean production of 975 ng/ml (range: 116–6763 ng/ml) to 87 ng/ml (range: 22–540 ng/ml) in the presence of αIL-21R (*p* = 0.004, Figure 4B). Taken together, these data show that the αIL-21R antibody has the capacity to inhibit B cell differentiation and subsequent immunoglobulin production.

## Discussion

We studied the activation of Tfh and memory B cells upon stimulation with alloantigen and the role of IL-21 within this process. The present study demonstrates that stimulation of Tfh and memory B cells with donor alloantigen results in an activated Tfh2 and Tfh17 phenotype, CSR of memory B cells, and differentiation of antigen-driven memory B cells toward IgM and IgG producing plasmablasts. In the presence of αIL-21R, formation of plasmablasts and IgM and IgG2 production were significantly inhibited. Our *in vitro* system is of high value to test other interventions that might alter Tfh-B cell interaction after alloantigen response.

While αIL-21R nicely inhibited IL-21 dependent STAT3p, the Tfh cell phenotype was not changed (Figure S5 in Supplementary Material). This suggests that the autocrine effect of IL-21 on the Tfh cell and the presence of antigen is not essential for upregulation of activation markers PD-1, ICOS, and chemokine receptors CCR6 and CXCR3. Our data are in line with *in vivo* and *in vitro* studies where the absence of either IL-21 or IL-6 did not affect Tfh differentiation, whereas combined absence of IL-21 and IL-6 led to a decreased Tfh frequency and Bcl6 gene expression (15, 30, 31). Thus, in our coculture system, the effect of IL-21 on the Tfh cell phenotype is redundant.

In our patient cohort, a high inter-individual variation in plasmablast numbers was observed after 8 days stimulation of Tfh and memory B cells with donor antigen (Figure 2B) reflecting the natural variation among patients. Response toward alloantigen apparently varied among the different cocultures, even though no significant differences in baseline characteristics were found between the cocultures with and without plasmablast formation (Table S1 in Supplementary Material). Immunological variation, e.g., distinct expression rates of co-stimulation surface markers or distinct IL-21 production by the Tfh cells among the different cocultures might contribute to this variation. Finally, effect of vaccination or viral infection might support the formation of alloantibody-producing plasmablasts *via* cross-reactivity. This cross-reactivity results in heterologous immunity within the patient population, this may contribute to the variation in plasmablast numbers in our cocultures (32).

In transplantation, the formation of complement fixing DSA and anti-HLA antibodies is associated with graft loss (2–5). In the presence of currently prescribed immunosuppressive drugs, *de novo* DSA and anti-HLA antibodies can be formed which contribute to the process of allograft rejection leading to graft loss (4, 33). A first hint that IL-21-producing Tfh cells are involved in processes leading to the production of alloantibodies comes from our study reporting that the absolute numbers of Tfh cells after transplantation are the highest in patients with pre-existing DSA (22). In addition, this study reports a decrease in plasmablast numbers when stimulating Tfh and memory B cells with staphylococcal enterotoxin B, a strong polyclonal superantigen (22). In the present study, we determined the mechanisms involved by analyzing the IL-21^+^ Tfh and B cell activation pathway during activation by donor antigen solely. This study demonstrates that alloantigen and IL-21 are key factors in this response. Translating the outcome of our *in vitro* study by using patient materials, we speculate that also *in vivo* B cell differentiation might be mediated by IL-21-producing Tfh cells and that interaction with donor antigen stimulated B cells results in the formation of DSA. Future experiments, e.g., *in vivo* experimental transplantation studies, should reveal whether indeed IL-21 drives antibody-mediated allogeneic immune responses.

In a phase I trial in healthy volunteers, 76% of the participants who received αIL-21R developed anti-drug antibodies due to increased activity of the antigen presentation machinery (34, 35). Thus, identification of an IL-21R blocker with lower immunogenicity or a switch to an IL-21 cytokine antagonist may be an alternative. In addition, in sensitized patients αIL-21R may not work sufficiently since these patients have high levels of circulating DSA due to previous transplants or pregnancy. In these patients, the destructive effects of the existing plasma cells need to be neutralized, for instance *via* protease inhibitors, inhibition of IL-6, or inhibition of BAFF and APRIL (6). To date, B-cell depletion is achieved *via* treatment with the anti-CD52 antibody alemtuzumab, anti-thymocyte globulin (ATG), and anti-CD20 antibody rituximab. Compared to these treatments, IL-21R blocking therapy is of interest since it is a biological that mainly interferes with the Tfh-B cell crosstalk, saving the presence of T and B cells with a resting or regulatory phenotype. The use of anti-IL6R treatment to decrease the formation of anti-HLA antibodies has also been widely studied. The first study in kidney transplant patients and experimental transplant models showed that anti-IL6R treatment affected the proportion Tfh cells and reduced B cell differentiation toward IgG-producing plasmablasts (36–38). In these studies, no data were reported on anti-IL21R agents and a next step would be to compare the functionality of these biologicals. We speculate that inhibition of both pathways, i.e., IL-6 and IL-21, may be of interest to test.

In this study, we focused on the interplay between peripheral Tfh cells and B cells. Low numbers of IL-21 producing T cells are present in the circulation, compared to high numbers found in inflamed tissues (39). In transplantation, the presence of Tfh cells that co-localized with B cells in follicular-like structures was confirmed in kidney biopsies taken during acute cellular rejection, suggesting IL-21-mediated Tfh–B cell interaction in local tertiary lymphoid structures in the kidney allograft (22, 23). In depth analysis of these graft infiltrating Tfh and B cells, e.g., *via* next generation sequencing or other single cell analysis, would be of great importance to improve our knowledge about molecular pathways involved in the anti-donor response.

Identifying the mode of action of Tfh-mediated activation of B cells upon alloantigen stimulation is important to further understand immunological processes that occur in transplant patients. In our allogeneic coculture system, IL-21 plays a non-redundant role in promoting B cell differentiation, as blockade of the IL-21/IL-21R signaling pathway resulted in almost complete inhibition of plasmablast formation as well as antibody production. In conclusion, our results demonstrate that IL-21 produced by alloantigen activated Tfh cells controls B cell differentiation toward antibody producing plasmablasts. The IL-21R might, therefore, be a useful target in organ transplantation to prevent rejection.

## Author Contributions

KL contributed to data generation, data analysis, and preparation of the manuscript. MD contributed to data generation and data analysis and reviewed the manuscript. FD, LL, RH, and CB contributed to the research design and preparation of the manuscript and reviewed the manuscript.

## Conflict of Interest Statement

The authors of this manuscript have no conflicts of interest to disclose related to this manuscript.

## Abbreviations

ADA, anti-drug antibodies; ATG, anti-thymocyte globulin; Bcl-6, B-cell lymphoma 6; CSR, class switch recombination; CXCR5, C–X–C chemokine receptor 5; DSA, donor-specific antibodies; GC, germinal center; HLA, human leukocyte antigen; ICOS, inducible co-stimulator; Ig, immunoglobulin; IL-21R, interleukin 21 receptor; PBMC, peripheral blood mononuclear cells; PD-1, programmed death 1; SEB, staphylococcal enterotoxin B; Tfh cell, follicular T helper cell.
