# Supplementary material for: IL-21 Receptor Antagonist Inhibits Differentiation of B Cells toward Plasmablasts upon Alloantigen Stimulation
Source: Front Immunol. 2017 Mar 20;8:306. doi: 10.3389/fimmu.2017.00306 (PMC5357809; doi:10.3389/fimmu.2017.00306)
Supplement: Supplementary file 1 [file Data_Sheet_1.pdf]

Title: IL-21R antagonist inhibits differentiation of B cells toward plasmablasts upon alloantigen stimulation

Running title: IL-21 and Tfh-B cell interaction

Kitty de Leur[1,2], Frank J.M.F. Dor[2], Marjolein Dieterich[1], Luc J.W. van der Laan[2], Rudi W. Hendriks[3], Carla C. Baan[1]

[1]Department of Internal Medicine, [2]Department of Surgery, Division of HPB & Transplant Surgery, [3]Department of Pulmonary Medicine, Erasmus MC, University Medical Center, Rotterdam, The Netherlands

Corresponding author:
Kitty de Leur
k.deleur.1@erasmusmc.nl

Supplemental figures

Supplemental Figure 1.

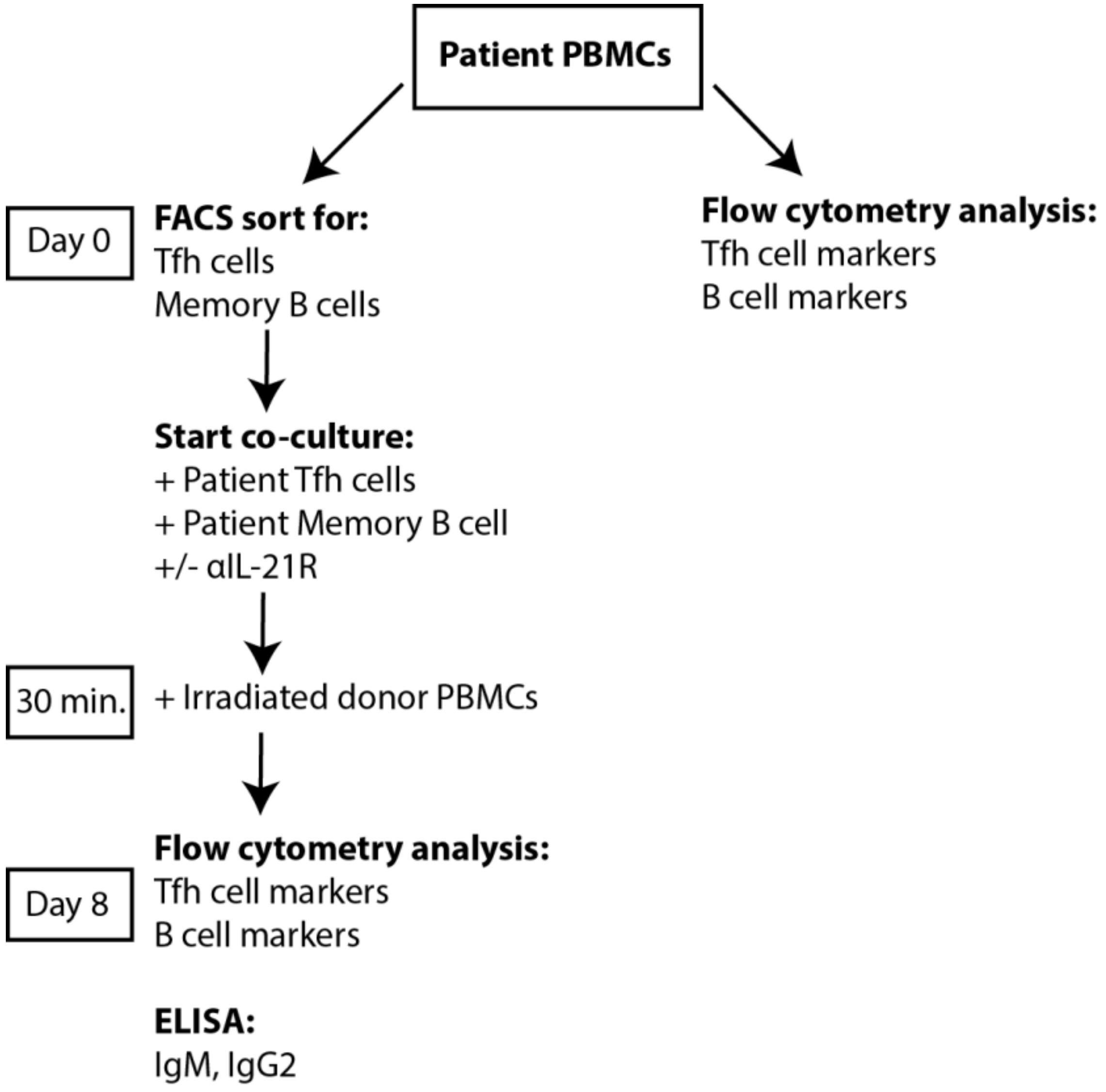

Supplemental Fig. 1.
Schematic representation of the co-culture model and analytical approaches used in this study.

Supplemental Figure 2.

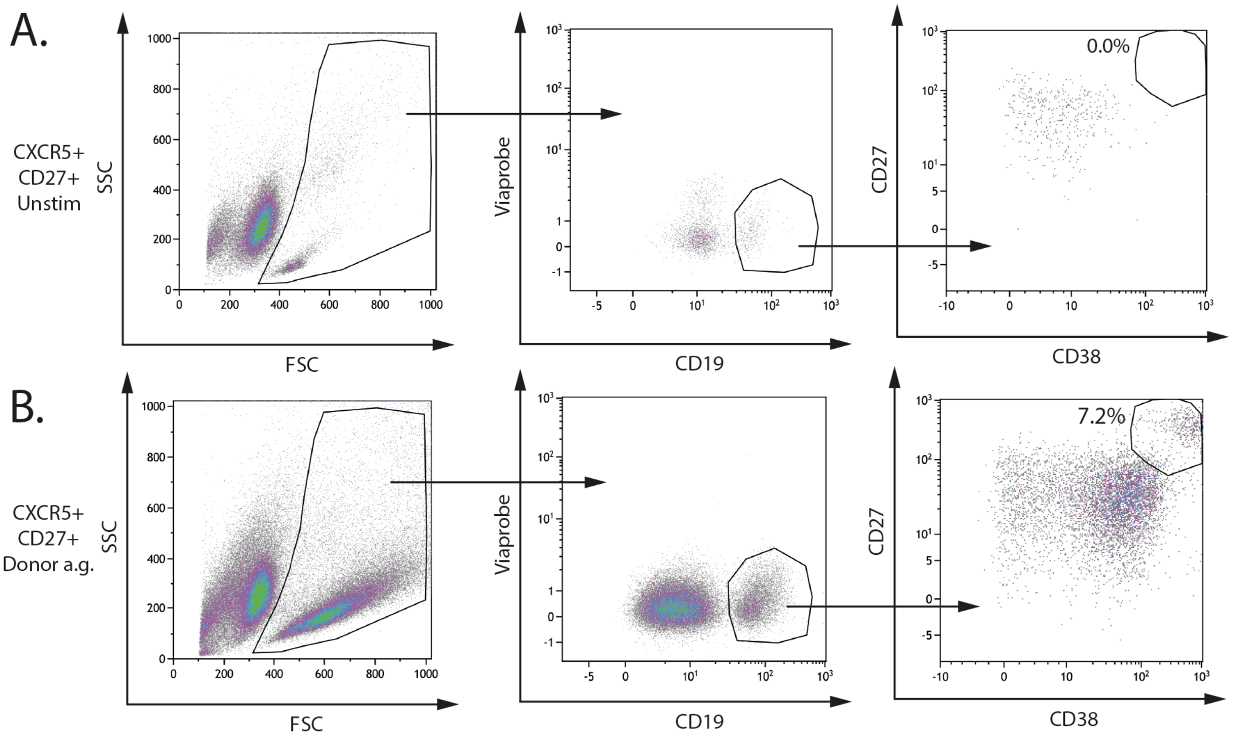

Supplemental Fig. 2.
CD4^{pos}CXCR5^{pos} Tfh cells and CD19^{pos}CD27^{pos} memory B-cells were FACS sorted and cultured for 8 days in the absence (A) or presence (B) of donor antigen. Cells were gated by forward- and side-scatter and viability was determined by 7-AAD negative cells (viaprobe). Plasmablasts (CD27^{high}CD38^{high}) were gated from the total B cell population (CD19^{pos}).

Supplemental table 1.

	Plasmablast formation:		
	**No (n=8)**	**Yes (n=9)**	***p* value (two-sided)**
**Patients age in years (median, range)**	50 (33-68)	62 (39-74)	0.89
**Recipient gender (% male)**	87.5%	66.7%	0.58
**HLA-A mismatches (mean ± s.d.)**	1,1 (±0.6)	1.0 (±0.9)	0.83
**HLA-B mismatches (mean ± s.d.)**	1.6 (±0.5)	1.7 (±0.5)	0.33
**HLA-DR mismatches (mean ± s.d.)**	1.5 (±0.5)	1.6 (±0.5)	0.35
**Panel reactive antigen (median, range)**			
**• Current**	8.9% (0.0-71%)	1.1% (0.0-5%)	0.80
**• Peak**	14,9% (0.0-99%)	1.7% (0.0-4%)	0.23
**Previous kidney-transplantation**	0% (0)	22.2% (2)	
**• Second kidney transplantation**	-	11.1% (1)	
**• Third kidney transplantation**	-	11.1% (1)	
**Renal replacement therapy before transplantation**			
**• None**	0% (0)	22.2% (2)	0.36
**• Haemodialysis**	87.5% (7)	66.7% (6)	
**• Peritoneal dialysis**	12.5% (1)	11.1% (1)	

Baseline characteristics of co-cultures with plasmablast formation (>10%) and without plasmablast formation (<5%). Numbers between brackets represent patient number unless otherwise specified

Supplemental Figure 3.

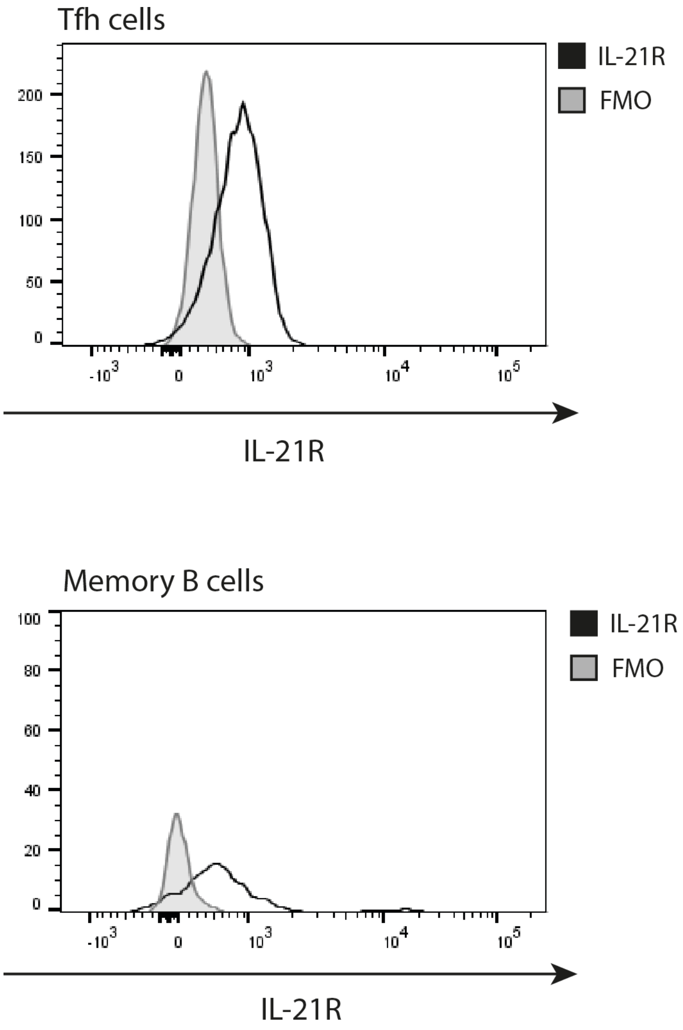

Supplemental Fig. 3.
Histogram overlays of IL-21R MFI values (black) or FMO (grey) on Tfh cells and memory B-cells. MFI: mean fluorescence intensity, FMO: fluorescence minus one.

Supplemental Figure 4.

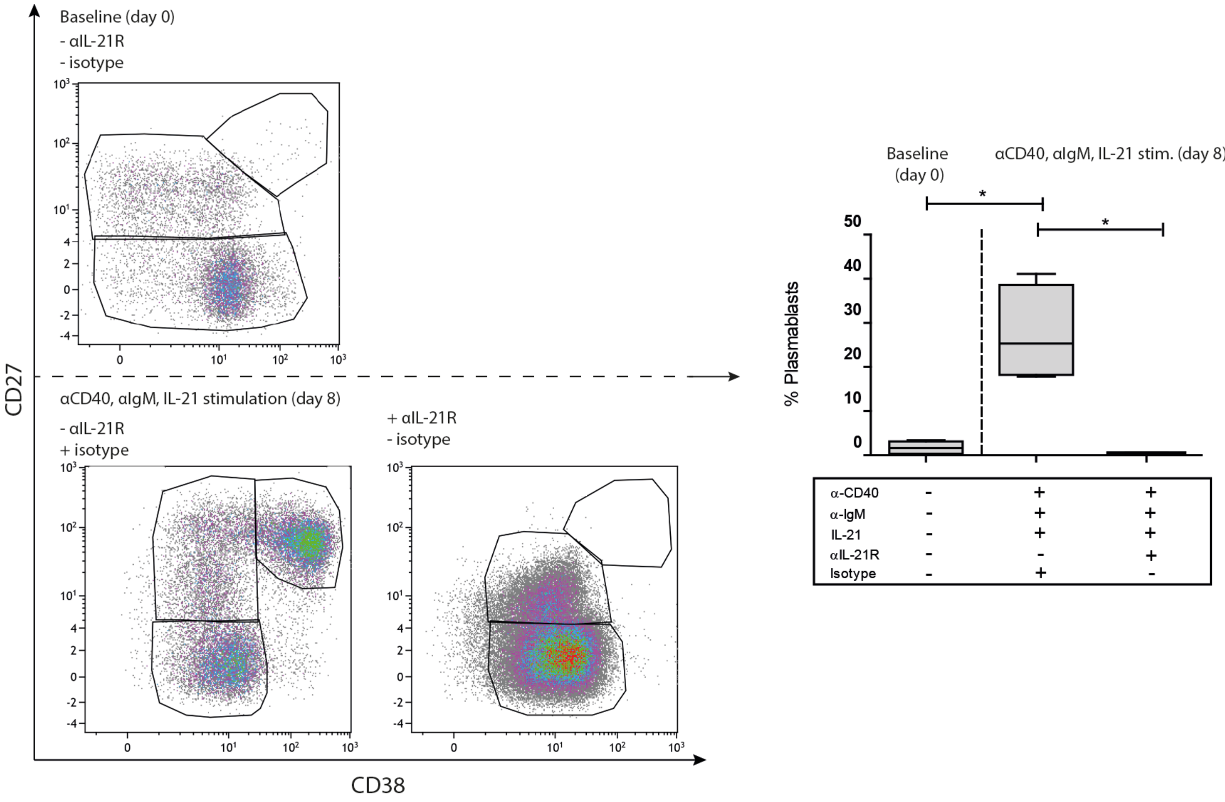

Supplemental Fig. 4.
CD19^{pos} B cells were stimulated with 5ug/ml α-CD40, 10ug/ml α-IgM and 100 ng/ml IL-21 for 8 days in the presence of 10ug/ml αIL-21R or 10ug/ml IgG1 isotype. Representative dotplots and quantified data of CD27^{high}CD38^{high} plasmablasts proportions at day 0 (baseline), and day 8 are shown. N.B.: Box whiskers represent minimal and maximal values. The upper and lower border of the box represent the 25% and 75% percentile with the middle line representing the median (n=4). *p<0.03.

Supplemental Figure 5.

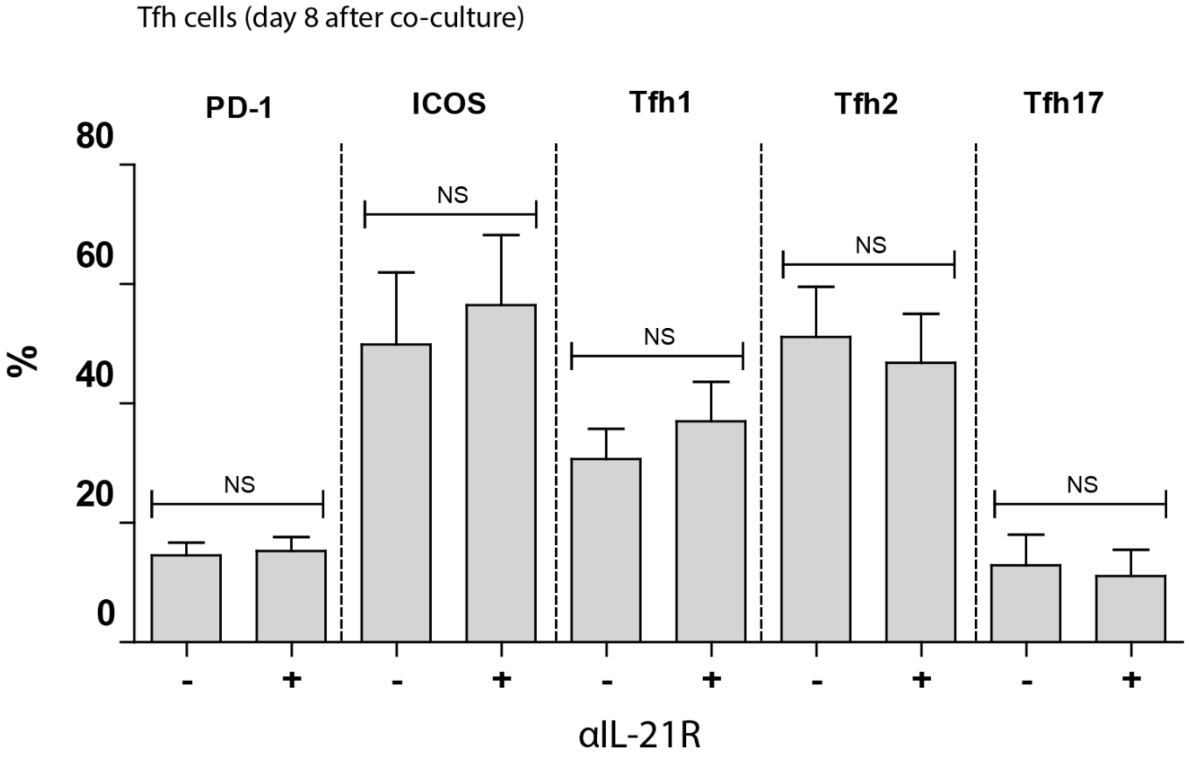

Supplemental Fig. 5.
Tfh and memory B-cells were co-cultured for 8 days in the presence (+) or absence (-) of 10ug/ml αIL-21R. (A) Percentages of PD-1^{pos}, ICOS^{pos}, CCR6^{neg}CXCR3^{pos} Tfh1, CCR6^{neg}CXCR3^{neg} Tfh2 and CCR6^{pos}CXCR3^{neg} Tfh17 cells within the Tfh population after 8 days co-culture. Upper line of the boxes represent mean with SEM represented by the whiskers. n=8, NS=not significant.
